# Atrial fibrillation is associated with increased in-hospital mortality and complications after IV t-PA in acute ischemic stroke: evidence from the China Stroke Center Alliance (CSCA)

**DOI:** 10.3389/fneur.2025.1667855

**Published:** 2026-01-14

**Authors:** Liwen Xie, Ruhui Liu, Zhigang Liang, Minghui Du, Hanye Yuan, Tianhao Zhang, Zhuqing Luan, Zhongwen Sun, Kaixuan Yang

**Affiliations:** 1 Department of Neurology, Yantai Yuhuangding Hospital Affiliated to Qingdao University, Yantai, China; 2 The Second Clinical Medical College, Binzhou Medical University, Yantai, China; 3 China National Clinical Research Center for Neurological Diseases, Beijing Tiantan Hospital, Capital Medical University, Beijing, China

**Keywords:** atrial fibrillation, cerebral hemorrhage, in-hospital mortality, intravenous thrombolysis, stroke

## Abstract

**Background and purpose:**

Regarding the prognosis of patients with acute ischemic stroke (AIS) complicated by atrial fibrillation (AF) after thrombolysis, previous studies have reported conflicting results. This research investigates the connection between AF and in-hospital mortality following intravenous thrombolysis (IVT) in individuals with AIS.

**Methods:**

Patient data were obtained from the Chinese Stroke Center Alliance (CSCA). This study constitutes a multicenter, retrospective cohort analysis, focusing on patients who received IVT using t-PA following an AIS. The primary outcome is adverse functional outcomes, characterized by in-hospital mortality. Safety outcomes mainly include cerebral hemorrhage. Univariate and multivariate logistic regression analyses were used to evaluate the relationship between AF and IVT outcomes.

**Results:**

A total of 48,294 patients participated in the study, including 5,465 with a history of AF. Baseline characteristics indicated that patients in the AF group were of advanced age at the time of onset (74 years vs. 65 years, *p* < 0.001). The NIHSS score at admission was higher (11 vs. 5, *p* < 0.001), and a modified Rankin Scale (mRS) score ≥3 before admission was more common (28.7% vs. 24.8%, *p* < 0.001). Regarding safety results, patients suffering from AF had higher in-hospital mortality (3.1% vs. 0.8%; adjusted OR 1.77, 95% CI 1.41–2.23).

**Conclusion:**

The study highlights that having a past of AF is linked to a higher chance of cerebral hemorrhage and in-hospital mortality in Chinese AIS patients following IVT. AF history is a strong predictor of in-hospital mortality (AUC = 0.85).

## Introduction

Ischemic stroke accounts for 82.6% of all strokes in China, making it the primary cause of disability-adjusted life years and death (1). Intravenous delivery of tissue plasminogen activator (t-PA) has demonstrated enhancements in clinical outcomes for individuals experiencing acute ischemic stroke (AIS) (2, 3). Atrial fibrillation (AF) is a risk factor for stroke that increases the likelihood of having one by almost five times (4). Survivors of AIS often experience major adverse cardiovascular events (MACE) within 2 years, with the presence of AF further heightening the risk of MACE following a stroke (5).

Can AIS patients with AF safely undergo thrombolysis and experience substantial benefits from this treatment? Opinions on this subject remain divided. It is worth noting that in patients with acute ischemic stroke and atrial fibrillation, early recurrent embolization has been identified as one of the most important predictors for in-hospital mortality (6), underscoring the potential severity of AF-related strokes. Kazumi et al. indicated that AF was independently linked to insufficient early recanalization following t-PA treatment (7). Additionally, a history of AF has been linked to early ischemic stroke recurrence following intravenous thrombolysis (IVT) (8). However, Brown et al. discovered that there was no correlation between atrial fibrillation (AF) and significant neurological improvement within 24 h following the administration of t-PA (9). In contrast, other studies suggest that AIS patients with AF who undergo IVT may experience fewer adverse outcomes than those without AF (10, 11).

Philipp et al. conducted a prospective randomized controlled trial (RCT) and found that patients taking direct oral anticoagulants (DOACs) did not develop symptomatic intracerebral hemorrhage (ICH) following IVT with alteplase, even beyond approved indications (12).

We hypothesize that AIS patients with a history of AF respond less favorably to t-PA treatment compared to those without AF. This research seeks to investigate the traits, in-hospital mortality rates, and complications associated with AIS patients who received t-PA treatment, both with and without a prior history of AF, utilizing registry data from the China Stroke Center Alliance (CSCA).

## Methods

The data used in this study were sourced from the China Stroke Center Alliance (CSCA) database, a nationwide, multicenter, prospective, continuous quality improvement initiative. Written informed consent was obtained from all patients or their legal surrogates at the time of enrollment into the CSCA database. Furthermore, the specific protocol for this study was reviewed and approved by the Ethics Committee of Beijing Tiantan Hospital, Capital Medical University. All statistical analyses were performed using de-identified data to protect patient privacy (13).

### Patient characteristics

Data on patients was gathered from all hospitals within the CSCA network from January 2018 to October 2023. The criteria for inclusion were determined: (1) Patients aged 18 years or older; (2) Diagnosis of AIS based on WHO standards, confirmed by brain computed tomography (CT) or magnetic resonance imaging (MRI); (3) Receipt of IVT with t-PA within 4.5 h of symptom onset in China (14); (4) Admission can occur either directly or via the emergency department. Patients were excluded: (1) Missing data, such as information on complications, in-hospital mortality, or AF status; (2) Patients undergoing endovascular treatment (EVT) after IVT.

### Clinical outcome measurement

The main focus was on measuring in-hospital mortality as the primary outcome, defined as a poor functional result. Secondary outcomes included recurrent stroke, cerebral hemorrhage, pneumonia, urinary tract infection, deep vein thrombosis, depression, seizure, and cardiac or respiratory arrest.

### Statistical analysis

Continuous variables were expressed as interquartile ranges (IQR), while categorical variables were shown as percentages. Baseline characteristics were assessed using Wilcoxon rank-sum tests for continuous variables and χ^2^ tests for categorical variables to compare differences. Odds ratios (ORs) and their 95% confidence intervals (CIs), including adjusted ORs, were calculated through univariate and multivariate logistic regression analyses. Adjustments were made for variables including mortality, stroke recurrence, cerebral hemorrhage, pneumonia, urinary tract infection, deep vein thrombosis, depression, seizure, and cardiac or respiratory arrest. Statistical analyses were conducted utilizing SAS 9.4, with a significance level set at *p* < 0.05.

## Result

**Figure 1:**
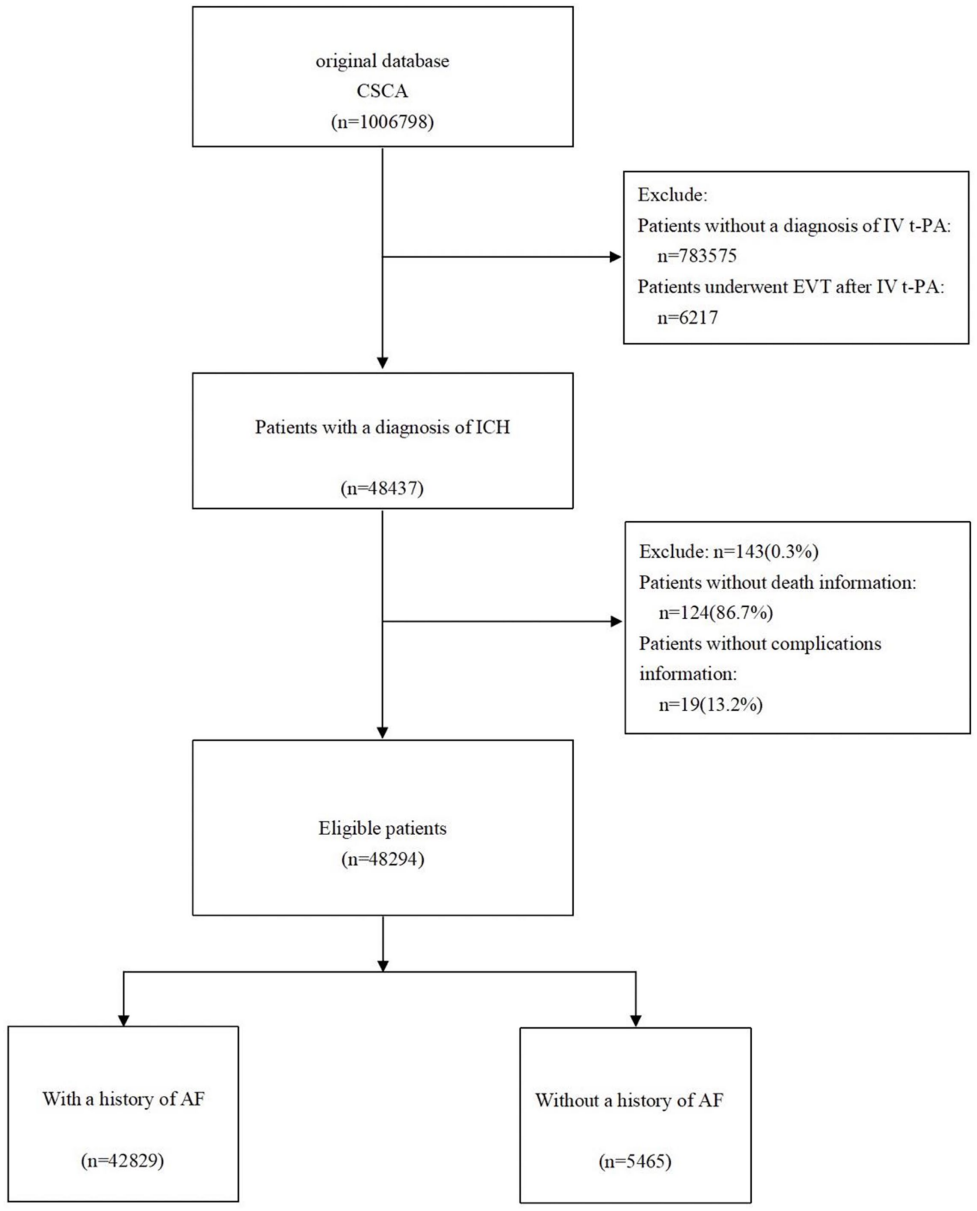
Study flowchart.

Initially 1,006,798 patients were screened from the CSCA database. After excluding 168,569 patients without a diagnosis of AIS, 783,575 patients without IV t-PA treatment, and 6,217 patients who underwent EVT following IV t-PA, 143 patients with missing death information, and 19 patients without complication data, adding up to 48,294 patients with AIS who received IV thrombolysis were included in the final analysis. Of these, 16,759 (34.7%) were women. The patient selection process is outlined in Figure 1.

### Baseline characteristics of patients

Table 1, Baseline table, summarizes the demographic and clinical profiles of patients. Patients suffering from AF tended to be older, were more inclined to smoke, and had higher rates of prior stroke, transient ischemic attack (TIA), coronary heart disease (CHD), myocardial infarction (MI), heart failure, chronic obstructive pulmonary disease (COPD), and peripheral vascular disease compared to those without AF. They were also more likely to have a pre-admission Modified Rankin Scale (MRS) score of ≥3. Conversely, patients with AF had lower systolic blood pressure, consumed less alcohol, and had a lower prevalence of diabetes.

**Table 1:** Baseline characteristics of patients with or without history of AF.

Variables	Total (*n* = 48,294 [100%])	Without history of AF (*n* = 42,829 [88.7%])	With history of AF (*n* = 5,465 [11.3%])	*p* Value
Demographic				
Age, years (IQR)	66.0 (57.0–74.0)	65.0 (56.0–73.0)	74.0 (67.0–80.0)	<0.001
Male	31,535 (65.3)	28,766 (67.2)	2,769 (50.7)	<0.001
Physical examination				
BMI	23.4 (21.1–25.5)	23.4 (21.2–25.6)	22.9 (20.5–25.1)	<0.001
Systolic blood pressure, mmHg (IQR)	150.0 (136.0–168.0)	151.0 (137.0–168.0)	148.0 (134.0–164.0)	<0.001
Diastolic blood pressure, mmHg (IQR)	87.0 (79.0–97.0)	87.0 (79.0–97.0)	87.0 (78.0–97.0)	0.33
Behavioral history				
Current smoking, *n* (%)	28,039 (58.1)	24,235 (56.6)	3,804 (69.6)	<0.001
Drinking, *n* (%)	12,507 (25.9)	11,514 (26.9)	993 (18.2)	<0.001
Medical history				
Prior stroke or transient ischemic stroke, *n* (%)	12,641 (26.2)	10,751 (25.1)	1890 (34.6)	<0.001
Hypertension, *n* (%)	29,019 (60.1)	25,660 (59.9)	3,359 (61.5)	0.03
Diabetes mellitus, *n* (%)	8,595 (17.8)	7,711 (18.0)	884 (16.2)	<0.001
Dyslipidemia, *n* (%)	3,238 (6.7)	2,828 (6.6)	410 (7.5)	0.01
Prior coronary heart disease or myocardial infarction, *n* (%)	4,747 (9.8)	3,620 (8.5)	1,127 (20.6)	<0.001
Heart failure, *n* (%)	700 (1.4)	312 (0.7)	388 (7.1)	<0.001
Chronic obstructive pulmonary disease, *n* (%)	598 (1.2)	449 (1.0)	149 (2.7)	<0.001
Peripheral vascular disease, *n* (%)	668 (1.4)	506 (1.2)	162 (3.0)	<0.001
Treatment				
Antiplatelet agents, *n* (%)	8,045 (16.7)	6,502 (15.2)	1,543 (28.2)	<0.001
**Anticoagulant agents, *n* (%)**	1830 (3.8)	1,096 (2.6)	734 (13.4)	<0.001
Antihypertensive agents, *n* (%)	20,358 (42.2)	17,753 (41.5)	2,605 (47.7)	<0.001
Antidiabetic agents, *n* (%)	6,513 (13.5)	5,817 (13.6)	696 (12.7)	0.08
Chinese patent drug, *n* (%)	2,930 (6.1)	2,393 (5.6)	537 (9.8)	<0.001
Scales and laboratory result				
Baseline modified ranking scale >3, *n* (%)	12,180 (25.2)	10,613 (24.8)	1,567 (28.7)	<0.001
**NIHSS score at admission (IQR)**	6.0 (3.0–11.0)	5.0 (3.0–10.0)	11.0 (5.0–16.0)	<0.001
Fasting blood glucose, mmol/L (IQR)	5.8 (5.0–7.1)	5.8 (5.0–7.1)	5.9 (5.1–7.2)	<0.001
Low-density Lipoprotein, mmol/L (IQR)	2.7 (2.1–3.3)	2.7 (2.1–3.4)	2.4 (1.9–3.0)	<0.001
Hemocysteine, (IQR)	13.3 (10.0–18.7)	13.3 (10.0–18.7)	13.6 (10.2–18.3)	0.30
Blood platelet, (IQR)	201.0 (161.0–244.0)	204.0 (165.0–246.0)	178.0 (142.0–218.0)	<0.001
Other information				
Onset to IVT, min (%)				<0.001
0–3 h, *n* (%)	28,859 (60.1)	25,441 (59.7)	3,418 (62.9)	
3–4.5 h, *n* (%)	17,904 (37.3)	15,997 (37.5)	1907 (35.1)	
>4.5 h, *n* (%)	1,277 (2.7)	1,166 (2.7)	111 (2.0)	

BMI, Body Mass Index; NIHSS, National Institute of Health Stroke Scale; IVT, intravenous thrombolysis.

Regarding treatment, 13.4% (734/5,465) of patients with AF were prescribed oral anticoagulants compared to 2.6% (1,096/42,829) of those without AF. Antiplatelet agents were used in 15.2% (650/5,465) of patients with AF and 28.2% (1,543/42,829) of patients without AF. Patients who did not have AF were more likely to undergo thrombolysis after a time period exceeding 4.5 h from symptom onset.

To further delineate the specific cardiac disorders in AIS patients with AF, we referred to the study by Pujadas-Capmany et al. (15). In their cohort of 402 cardioembolic stroke patients, the most frequent etiology was hypertrophic hypertensive heart disease complicated with AF (29.8%), followed by isolated AF (21.9%), left ventricular systolic dysfunction (22.6%), and rheumatic mitral valve disease (12.4%). Although systematic transthoracic echocardiography data were not available for all patients in our cohort, our AF patients exhibited a higher prevalence of heart failure, coronary heart disease, and peripheral vascular disease, indirectly suggesting an underlying burden of structural heart disease. These findings collectively underscore the importance of identifying and managing specific cardiac abnormalities in stroke patients with AF for comprehensive management and secondary prevention.

### Safety outcomes

Regarding the safety outcomes presented in Table 2 (safe outcome), after adjusting for variables such as age, sex, hypertension, diabetes mellitus, heart failure, history of stroke or transient ischemic attack (TIA), coronary heart disease (CHD) or myocardial infarction (MI), chronic obstructive pulmonary disease (COPD), smoking, alcohol consumption, LDL-C, fasting plasma glucose (FPG), homocysteine (HCY), platelet count (PLT), NIHSS score, mRS score, and other factors, the in-hospital mortality rate was 3.1% (170/5465) among patients who have previously been diagnosed with atrial fibrillation(AF), compared to 0.8% (359/42829) among those without AF (crude OR 3.89; 95% CI 3.23–4.69). Following adjustment for multiple variables, the statistical significance of this difference persisted (adjusted OR 1.77; 95% CI 1.41–2.23).

**Table 2:** Rate of in-hospital mortality and complications in IV t-PA treated AIS patients with VS. without a history of AF.

Outcome	With history of AF [Event/*N* (%)]	Without history of AF [Event/*N* (%)]	Unadjusted		Multivariable adjusted*	
			OR (95%CI)	*p* value	OR (95%CI)	*p* value
Mortality	170/5465 (3.1)	359/42829 (0.8)	3.89 (3.23, 4.69)	<0.001	1.77 (1.41, 2.23)	<0.001
Complication						
Stroke recurrence	310/5465 (5.7)	1934/42829 (4.5)	1.27 (1.13, 1.44)	<0.001	1.00 (0.86, 1.15)	0.953
Cerebral hemorrhage	472/5465 (8.6)	1160/42829 (2.7)	3.46 (3.09, 3.87)	<0.001	2.15 (1.88, 2.46)	<0.001
Pneumonia	1425/5465 (26.1)	4602/42829 (10.7)	2.98 (2.78, 3.19)	<0.001	1.48 (1.36, 1.61)	<0.001
Urinary tract infection	154/5465 (2.8)	553/42829 (1.3)	2.25 (1.88, 2.70)	<0.001	1.29 (1.04, 1.59)	0.020
Deep vein thrombosis	127/5465 (2.3)	518/42829 (1.2)	1.98 (1.63, 2.41)	<0.001	1.32 (1.04, 1.66)	0.020
Depression	104/5465 (1.9)	607/42829 (1.4)	1.36 (1.11, 1.68)	<0.001	1.16 (0.91, 1.47)	0.237
seizure	72/5465 (1.3)	237/42829 (0.6)	2.39 (1.83, 3.12)	<0.001	1.46 (1.07, 2.01)	0.018
Cardiac or respiratory arrest	139/5465 (2.5)	273/42829 (0.6)	4.15 (3.38, 5.11)	<0.001	1.77 (1.38, 2.29)	<0.001

*Adjusted for Abbreviations: mRS, Modified Ranking Score; OR, Odds Ratio; SD, Standardized Deviation; CI, Confidence Interval.

In terms of complications, the incidence of cerebral hemorrhage during hospitalization was 8.6% (472/5465) in patients with a history of AF compared to 2.7% (1,160/42829) in those without AF. Statistical analysis demonstrated significant differences between the two groups (adjusted OR 2.15; 95% CI 1.88–2.46). Additionally, patients who have previously received a diagnosis of AF demonstrated a higher likelihood of developing pneumonia (adjusted OR 1.48; 95% CI 1.36–1.61) and cardiac or respiratory arrest (adjusted OR 1.77; 95% CI 1.38–2.29).

**Figure 2:**
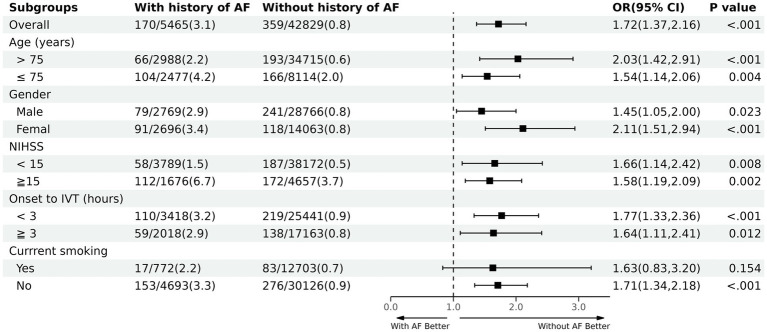
Subgroup analysis. NIHSS, National Institute of Health Stroke Scale; IVT, intravenous thrombolysis.

Subgroup analyses (Figure 2) explored the relationship between age, sex, NIHSS on admission, time from symptom onset to IV thrombolysis (IVT), and current smoking. These analyses revealed that, except for current smoking, patients diagnosed with AF consistently exhibited a higher in-hospital mortality rate. Notably, among patients without a history of AF, those over 75 years old (crude OR 2.03; 95% CI 1.42–2.91) and patients who are female (crude OR 2.11; 95% CI 1.51–2.94) derived significant benefits from IVT.

### Predictive factors outcomes

**Figure 3:**
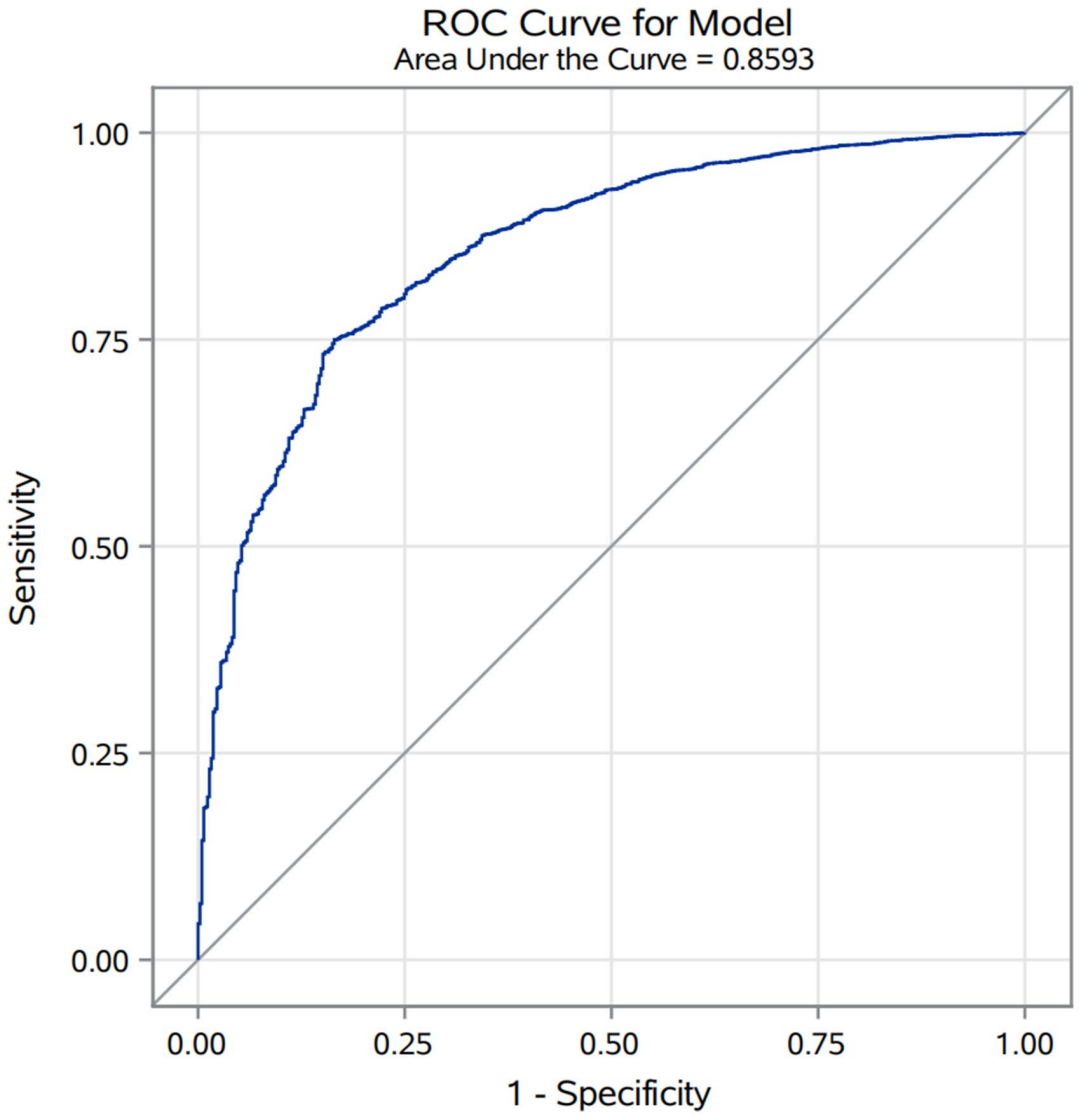
ROC curve of atrial fibrillation history for predicting in-hospital mortality in acute ischemic stroke patients after intravenous thrombolysis.

Logistic regression analysis identified a history of AF as a predictive factor for in-hospital mortality in AIS patients after thrombolysis. The factor’s predictive accuracy was evaluated through the area under the receiver operating characteristic (ROC) curve. It achieved an AUC of 0.859, along with a sensitivity of 74.9% and a specificity of 83.5% (refer to Figure 3). This suggests that AF is a reliable predictor of in-hospital mortality following thrombolysis, with substantial clinical utility.

## Discussion

This study, involving nearly 50,000 patients, highlights that approximately 8.6% of individuals with a history of AF experience cerebral hemorrhage following intravenous thrombolysis. While the specific causes of in-hospital death (e.g., neurological vs. non-neurological) were not available for analysis, the significantly higher rates of cerebral hemorrhage, pneumonia, and cardiac/respiratory arrest in the AF group (Table 2) nevertheless suggest that both neurological complications and systemic medical issues contribute to the elevated mortality observed. Moreover, a history of AF was identified as a strong predictor of in-hospital mortality after thrombolytic therapy. Patients over the age of 75 and female patients experienced particularly elevated mortality rates.

Globally, it is well-established that individuals with AF are at a heightened risk of both mortality and cerebral hemorrhage after thrombolytic therapy. The findings of this study align with those of previous research (7, 16). Additionally, TIAN et al. demonstrated through a line chart prediction model that patients with AF face an increased risk of early functional decline following intravenous thrombolysis for AIS (17). However, the underlying causes of these disparities remain incompletely understood.

Potential explanations include the following: Patients diagnosed with AF, the stroke type is frequently cardiogenic. The efficacy of IVT depends on several factors, including the thrombus’s location and size, the drug’s ability to penetrate it, and the time elapsed since the thrombus’s formation (16). Patients with AF often develop thrombi within the heart chambers, which become increasingly difficult to dissolve as they enlarge or persist over time. Prior studies have shown that AF patients often present with elevated baseline NIHSS scores, likely due to larger infarct sizes and impaired collateral circulation (11, 18). Our baseline characteristics align with this; patients with AF chosen in CSCA exhibit elevated NIHSS scores. Yet, elevated NIHSS scores might be linked to increased rates of bleeding, ultimately resulting in higher in-hospital mortality rates. Hans et al. performed a comparison study on stroke patients with and without AF using data from a multimodal magnetic resonance imaging (MRI) trial that incorporated echoplanar imaging with diffusion and perfusion assessments. Their findings indicated that AF is linked to more severe baseline hypoperfusion, leading to larger infarct sizes, an elevated risk of hemorrhagic transformation, and poorer clinical results (19).

We hypothesize that patients with a history of AF are more likely to be on anticoagulants compared to those with newly diagnosed AF. It is important to note that the analysis was conducted under the predetermined data agreement of the CSCA, which did not include variables such as AF subtype, duration, or adequacy of anticoagulation. Future research with access to these specific clinical details would be valuable in further elucidating the mechanisms behind our findings. This may lead to a higher risk of bleeding conversion during IVT while still on anticoagulants, ultimately resulting in an increased mortality rate. Therefore, it is expected that after undergoing IVT, patients with a history of AF will have a higher mortality rate compared to those newly diagnosed with the condition. However, a retrospective study has suggested that both newly diagnosed and previously diagnosed AF are linked to the risk of rICH, with newly diagnosed patients being more susceptible to rICH than those with a history of the condition. The possible reasons for this include the higher likelihood of asymptomatic cerebral infarction in newly diagnosed AF patients compared to those with a history of AF. Additionally, newly diagnosed AF patients may have experienced AF previously but remained undiagnosed due to the absence of an electrocardiogram examination. Asymptomatic AF patients are less likely to be detected, and no AF waveform may appear during electrocardiogram evaluations. Furthermore, newly diagnosed AF patients are unlikely to be prescribed medications such as antiplatelet, anticoagulant, or statin (20) lipid-lowering therapy (21). rICH can result in severe consequences, particularly an increased in-hospital mortality rate. Based on the research mentioned above, it is speculated that the higher in-hospital mortality rate among patients with AF undergoing IVT is more likely attributed to AF itself, rather than a high NIHSS score.

Our investigation also revealed that gender disparities are associated with outcomes following thrombolysis. The systematic review by Appelros et al. demonstrated that women, despite having a lower overall incidence of stroke, are more severely affected, presenting at an older age and experiencing higher one-month case fatality. This finding is corroborated by other studies which also indicate that the global burden of stroke, in terms of severity, is consistently greater in women than in men (22–24). Recent studies have shown that younger women with ischemic stroke have lower incidence rates than younger men, while older women have higher rates compared to older men (25). The antithrombotic properties of endogenous estrogen are believed to be responsible for this trend. Zhou et al. (26) discovered that women tend to benefit more from treatment than men in cases of ischemic stroke, a finding that aligns with our observations.

Considering the potential risks associated with newly diagnosed AF and a history of AF in patients with AIS, it is imperative to enhance awareness and implement appropriate strategies for AF detection (27). Community hospitals can perform electrocardiogram screenings for AF in residents and provide education on the risks associated with AF-related strokes (27). Targeting high-risk groups for stroke recurrence, such as patients with cryptogenic stroke, implantable cardiac monitoring devices (ICMs) can be used (28) to identify occult AF. These devices offer diagnostic and preventive benefits for unexplained stroke.

Shimada et al. (29) revealed that the occurrence of premature atrial contractions (PACs) in individuals with cryptogenic stroke exhibits a dose-dependent relationship with the detection of atrial fibrillation, suggesting that patients with more frequent PACs are at an elevated risk of being diagnosed with AF. Furthermore, the STROKE AF study highlights that among patients diagnosed with AIS and categorized as having large artery atherosclerosis (LAD) or small vessel occlusive disease (SVD), 12% developed AF within a year (30), as monitored by an implanted cardiac monitor (ICM). The inclusion of ICMs in routine testing is recommended for cases of unexplained stroke (31). When used as a secondary prevention measure, ICMs are particularly beneficial for patients who have congestive heart failure and left atrial enlargement (32).

Considering that patients who have AIS combined with AF have poorer IVT treatment results than those without AF (33), low-dose recombinant tissue plasminogen activator (rt-PA) (<0.85 mg/kg) may be considered to improve prognosis (34). Additionally, anticoagulant-treated AF patients undergoing endovascular thrombectomy (EVT) show better functional outcomes after 90 days, with a comparable rate of sICH. Administering thrombolysis prior to performing EVT in patients with atrial fibrillation has been shown to improve functional results at 90 days and reduce mortality rates (35). Q et al. (36) indicate that the combination of IVT and EVT enhances functional outcomes and reduces mortality rates in suffering from AIS caused by large vessel occlusion (AIS-LVO), particularly in those with AF. Declining IVT in conjunction with EVT could potentially be detrimental to patients with AF.

Conversely, Nogueira et al. (37) performed a combined analysis of data from two studies conducted in China and Japan. Three categories of Asian patients were examined: those with AF, patients experiencing the onset of EVT more than 180 min after symptom onset, and patients with occlusion of the intracranial internal carotid artery (ICA). The analysis suggested that employing EVT alone may result in superior outcomes compared to the combination of IVT and EVT. The optimal treatment approach for patients with AF in conjunction with AIS remains undetermined, necessitating further in-depth research.

Our study has several limitations. First, it only includes patient data from Chinese hospitals, which limits our ability to assess the safety variations of IVT in patients with AF and AIS across different ethnic groups. Moreover, the patients we recruited did not include those who underwent thrombectomy or bridging thrombolysis. Secondly, the analysis incorporates data from nearly 50,000 patients, which led to some missing data points within the database. Additionally, certain data, such as Modified Rankin Scale (MRS) scores and imaging information on the sizes and subtypes of stroke, were not reported in the CSCA project. This limitation restricts the scope of our prognostic data analysis. Lastly, as a retrospective analysis, we cannot completely eliminate the possibility of selection bias.

### Future research directions

While this study identified a history of AF as a significant predictor of in-hospital mortality following thrombolysis, future research should strive to identify earlier electrophysiological markers. Bayés syndrome, characterized by advanced interatrial block, is an under-recognized cardiac rhythm disorder with substantial clinical implications. Recent evidence has established it as a novel and independent risk factor for atrial fibrillation and cardioembolic stroke, and it may be responsible for a proportion of unexplained, cryptogenic strokes (38). Therefore, a promising and essential line of future inquiry would be to systematically evaluate the impact of Bayés syndrome as a predictor for incident atrial fibrillation and increased in-hospital mortality in patients with AIS. Investigating this association could facilitate the earlier identification of high-risk patients and potentially inform novel targeted prevention and intervention strategies.

## Conclusion

In China, we have found that AF is independently linked to cerebral hemorrhage in AIS patients and serves as a significant predictor of in-hospital mortality for those receiving IVT. To lower in-hospital mortality rates among AIS patients undergoing IVT, it is essential to implement community electrocardiogram screenings, monitor high-risk populations with ICMs, and promote standardized management approaches for strokes related to AF across the country. Moving forward, it is important to expand research variables and further investigate the best treatment strategies for patients with AIS who also have AF. Future studies should also consider the heterogeneity of AF (e.g., subtype, anticoagulation status), the role of cardiac monitoring in cryptogenic stroke, and the potential impact of Bayés syndrome on stroke mechanisms and outcomes.

## Data Availability

This study utilized data from the China Stroke Center Alliance (CSCA) database. The CSCA is a national, hospital-based, multicenter, voluntary, continuous quality improvement initiative. The data coordinating center is located at the China National Clinical Research Center for Neurological Diseases, Beijing Tiantan Hospital. Requests to access the datasets should be directed to the corresponding author.
